# Usefulness of Smartphones in Dermatology: A US-Based Review

**DOI:** 10.3390/ijerph19063553

**Published:** 2022-03-17

**Authors:** Samantha Ouellette, Babar K. Rao

**Affiliations:** 1 Department of Dermatology, Rutgers Robert Wood Johnson Medical School, Somerset, NJ 08873, USA; raobk@rwjms.rutgers.edu; 2 Department of Dermatology, Weill Cornell Medicine, New York, NY 10065, USA

**Keywords:** smartphone, application, apps, dermatology, AI, artificial, intelligence

## Abstract

(1) Background: As smartphones have become more widely used, they have become an appealing tool for health-related functions. For dermatology alone, hundreds of applications (apps) are available to download for both patients and providers. (2) Methods: The Google Play Store and Apple App Store were searched from the United States using dermatology-related terms. Apps were categorized based on description, and the number of reviews, download cost, target audience, and use of AI were recorded. The top apps from each category by number of reviews were reported. Additionally, literature on the benefits and limitations of using smartphones for dermatology were reviewed. (3) Results: A total of 632 apps were included in the study: 395 (62.5%) were marketed towards patients, 203 (32.1%) towards providers, and 34 (5.4%) towards both; 265 (41.9%) were available only on the Google Play Store, 146 (23.1%) only on the Apple App Store, and 221 (35.0%) were available on both; and 595 (94.1%) were free to download and 37 (5.9%) had a cost to download, ranging from USD 0.99 to USD 349.99 (median USD 37.49). A total of 99 apps (15.7%) reported the use of artificial intelligence. (4) Conclusions: Although there are many benefits of using smartphones for dermatology, lack of regulation and high-quality evidence supporting the efficacy and accuracy of apps hinders their potential.

## 1. Introduction

Over the years, smartphones have become extensions of ourselves, with the percentage of adults owning smartphones increasing from 35% to 85% between 2011 and 2021 [1]. As such, they have become the perfect target for health interventions, and lend themselves particularly well to dermatology, a largely visual specialty. Many of the diverse capabilities of smartphones come from the different applications (apps) available. The number of apps available has been increasing with advancements in the image quality that smartphones can attain [2]. Between 2014 and 2017, 235 new dermatology-related apps became available [3]. There is also a high rate of turnover for available apps; for example, a review of apps for prevention of skin cancer reported that of the 39 apps that were available in 2014, 30 were no longer available five years later in 2019 [4]. Here, we discuss the functions related to dermatology that can be carried out from a smartphone for both patients and providers.

## 2. Materials and Methods

The Apple App Store and Google Play Store were searched from 4 to 11 February 2022 from the United States (US) to identify the dermatology-related mobile apps available. The following search terms were used: “dermatology”, “mole”, “eczema”, “psoriasis”, “rosacea”, “acne”, “skin cancer”, “melanoma”, “hidradenitis suppurativa”, “UV”, “teledermatology”, “vitiligo”, and “dermoscopy”. Exclusion of apps included those pertaining to general medicine, private dermatology practices, entertainment, photograph editing, veterinary dermatology, claims to cure skin diseases, general cosmetic tips, tanning promotions, and apps without an English option available. From the remaining results, the dermatology-related apps and the corresponding number of consumer reviews were recorded, as the number of downloads is not available on the Apple App Store. Based on the description available for each app, apps were first categorized based off whether they were marketed to patients, providers, or both. Apps were further categorized based on similar categories used in a previous study in 2013 by Brewer et al.: “general dermatology reference”, “educational aid”, “disease guide”, “self-surveillance/diagnosis”, “teledermatology”, “Ultraviolet (UV) protection”, “calculator”, “conference”, “journal”, “photography”, and “research” [5]. Apps that did not fit into existing categories but had less than 5 apps per category were grouped under “other”. General weather apps that only displayed the UV index, without additional information provided about protection, were excluded. Only general telehealth apps that specifically mentioned dermatologists were included. The number of apps that use artificial intelligence (AI) or machine learning and the cost to download the app were recorded. Common features of each category are described based off the public descriptions provided. The top 5 apps from each category by number of reviews were listed for categories with 50 or more apps. The top 3 apps were listed for categories with 30–49 apps, the top 2 apps were listed for categories with 10–29 apps, and the top app was listed for categories with less than 10 apps.

## 3. Results

A total of 632 apps were included in the study: 395 (62.5%) were marketed towards patients, 203 (32.1%) were marketed towards providers, and 34 (5.4%) were marketed towards both; 265 (41.9%) were available only on the Google Play Store, 146 (23.1%) were available only on the Apple App Store, and 221 (35.0%) were available on both; and 595 (94.1%) were free to download and 37 (5.9%) had a cost to download, ranging from USD 0.99 to USD 349.99 (median USD 37.49). Of note, some apps require in-app purchases or subscriptions to use all the functions of the app, which were not assessed during this study. A total of 99 apps (15.7%) reported the use of artificial intelligence or machine learning. The characteristics of the top apps based on number of reviews for categories with 50 or more apps are listed in Table 1. The characteristics of the top apps based on number of reviews for categories with less than 50 apps are listed in Table 2. The developers of the apps mentioned in this study are listed in Appendix A, Table A1.

### 3.1. Disease Guide

The “disease guide” category had the most apps at 163 (25.8%). The top 5 apps in this category were MDacne—Custom Acne Treatment, Kopa by Happify Health, Acne Intelligence, Imagine Skin Condition Tracker, and myForte. Apps were placed in this category if they focused on specific diseases, such as acne, eczema, psoriasis, rosacea, hidradenitis suppurativa, or vitiligo. Often, these apps included information about the specific skin disease, such as etiology, pathogenesis, and treatments. A common function of these apps was disease monitoring, where patients could track severity, symptoms, triggers, and/or treatment response. Many of the apps that allowed disease monitoring had the option for users to add photos, with some reporting the ability to calculate percentage improvement over time based off AI analysis. Some of these apps graphed changes over time and allowed users to generate progress reports to share with their dermatologists. Other apps in this category used AI to analyze pictures of the skin to gauge disease severity and provide treatment recommendations. Few apps were primarily focused on linking users to social support groups for their condition or identifying ingredients in products that may exacerbate their condition.

### 3.2. Self-Surveillance/Diagnosis

The “self-surveillance/diagnosis” category was comprised of 94 apps (14.9%). The top 5 apps in this category were TroveSkin—Get Clearer Skin, Medgic—Scan, Analyze and Detect Skin Problems, Skin Bliss: Cosmetics & Beauty, Miiskin—Skin Cancer eHealth, and Skincare routine. Apps were included in this category if their primary focus was helping users autonomously monitor, diagnose, and treat their skin. The apps in this category that focused primarily on detecting skin cancer gave users the ability to either track their moles over time by logging pictures or utilize AI to generate a probability percentage that a mole is malignant. Often, these apps included an avatar to allow users to map the location of their moles, and reminders could be set at certain intervals for reimaging and comparison of photos. Some apps could also measure the size of a lesion using a reference object, such as a coin. Other apps in this category generated potential diagnoses for a skin condition based on AI analysis of a photo and/or a description provided by the user. Additionally, some apps in this category were focused on AI analysis of the skin to provide general product recommendations based on features such as wrinkles, dark spots, and hydration and allowed users to track their skin care routine.

### 3.3. Teledermatology

The “teledermatology” category was comprised of 70 apps (11.1%). The top 5 apps in this category were Teladoc|Telehealth & Therapy, Practo: Online Doctor Consultations & Appointments, MDLIVE: Talk to a Doctor 24/7, Lybrate: Consult Doctor Online, and Zocdoc. Apps were included in this category if their main function was to connect patients with dermatologists virtually. Apps either connected patients to their personal dermatologist or helped patients connect to a dermatologist for the first time. Some apps included video visits, while others allowed users to consult dermatologists by sending pictures and additional information about their conditions. Then, these dermatologists could provide diagnoses and treatment recommendations, as well as send prescriptions and recommend in-person visits if needed. These apps have varying geographical scopes, with some available only to residents of a specific state in the US and others providing access to dermatologists in all 50 states. Additionally, some apps provided teledermatology services internationally.

### 3.4. General Dermatology Reference

The “general dermatology reference” category was comprised of 62 apps (9.8%). The top 5 apps in this category were VisualDx, Dermatology Atlas & Skin Infections, Skin Disease and Treatment, Skin Diseases and Treatment, and Skin Disease Treatment Symptoms and Diagnosis 2019. This category included apps that functioned as sources of information about a variety of diseases, medications, and/or procedures. Additionally, some provided key features and representative photos of different diseases on dermoscopy or histopathology. Dermatology textbooks were also included in this category. Some of these apps had the option to use AI to provide a list of differential diagnoses based off information provided about a skin condition, ranging from a one-line case description to a comprehensive list of questions about information such as lesion type, location, symptoms, progression, and patient demographics.

### 3.5. Ultraviolet (UV) Protection

The “Ultraviolet (UV) protection” category was comprised of 62 apps (9.8%). The top 5 apps in this category were Weather data & microclimate: Weather Underground, Weather Home—Live Radar Alerts & Widget, UV index widget—worldwide, UV Index Now Forecast & Sun Tracker—UVI Mate, and UVLens—UV Index Forecasts. Many apps in this category displayed the local UV index and provided tips to protect users from the sun. Other common features of these apps were sunscreen recommendations and time to burn based off the user’s phototype, and the ability to set up reminders to reapply sunscreen throughout the day. Additionally, some apps allowed users to track total daily sun exposure, scan sunscreen barcodes to display if products are broad spectrum, SPF 30+, photostable, and water resistant, and determine how much sunscreen to wear based on the user’s size and clothes.

### 3.6. Educational Aid

The “educational aid” category was comprised of 57 apps (9.0%). The top 5 apps in this category were Dermatology by Dr. Manish Soni, Dermoscopy Two Step Algorithm, iDoc Academy, Skin Anatomy, and Top Derm: A game for dermatologists. Apps were included in this category if they focused on ways to reinforce learned material through flashcards, quizzes, and board examination preparation material. A subset of apps available were designed like games, with features such as level advancement, accumulation of point based on correct answers, and leader boards. Some apps also had the option to earn continuing medical education credits.

### 3.7. Conference

The “conference” category was comprised of 35 apps (5.5%). The top 3 apps in this category were AAD Meetings, Dermacon 2020, and Fall Clinical. The apps in this category were designed for specific dermatology conferences and annual meetings. They often included information on conference schedules, locations, and speakers.

### 3.8. Calculator

The “calculator” category was comprised of 25 apps (4.0%). The top 2 apps in this category were Melanoma Calculator and Skin Lymphoma. Apps were included in this category if their only function was to provide calculations. This category included apps with clinical severity assessment tools such as the Psoriasis Area and Severity Index and the SCORing Atopic Dermatitis Index, as well as patient questionnaires such as the Dermatology Life Quality Index. Other apps calculated risk assessments for developing skin cancer, determined staging for melanoma, and provided predictions about prognosis. Few apps in this category were used to determine the amount of topical medication, such as corticosteroids, needed to cover a certain area of the body.

### 3.9. Photography

The “photography” category was comprised of 16 apps (2.5%). The top 2 apps in this category were Sklip and HumazeMD. Apps in this category had the primary function of taking, transferring, and storing photos. This includes clinical, dermoscopic, and trichoscopic photos. Some of the apps for storing dermoscopic photos were compatible with dermoscopy attachments for smartphones.

### 3.10. Journal

The “journal” category was comprised of 9 apps (1.4%). There were not enough reviews to stratify apps in this category. Apps in this category were designed for specific dermatology journals. They commonly allowed users to download, save, and share recent publications.

### 3.11. Research

The “research” category was comprised of 7 apps (1.1%). The top app in this category was PeDRA Research. Apps in this category were either used for tracking participants of research studies or connecting users to research organizations.

### 3.12. Other

The “other” category was comprised of 32 apps (5.1%). The top 3 apps in this category were Think Dirty, SkinSafe, and EZDERM. The most common functions of apps in this category were connecting physicians to dermatologists for consultations, controlling home phototherapy devices, checking ingredients in skincare products, connecting to fundraising events and advocacy groups, and providing the latest news in dermatology.

## 4. Discussion

### 4.1. Benefits and Advantages

The ability to address patients’ dermatologic needs using smartphones is especially important given the disparity in access to dermatologists globally [6]. The International Foundation for Dermatology has estimated that 3 billion individuals in 345 rural communities do not have adequate access to dermatologic care [7]. In India, there is only one dermatologist per 400,000 people, with most residing in urban areas [8]. Accessibility to dermatologists is similar in many other Asian and African countries [8]. In the U.S., the number of dermatologists per capita has increased by over 20% since the mid-90s; however, this increase has occurred disproportionately in urban areas [9,10]. This disparity affects patient outcomes negatively for those in more rural, underserved areas without easy access to dermatologists [11,12]. Delayed or missed skin cancer screenings can lead to more undetected skin cancer, including melanoma, the most lethal form of skin cancer with better prognosis when detected early [11,12]. Importantly, as the U.S. population ages, the need for access to dermatologists will continue to increase [13,14]. Furthermore, the rising incidence of skin cancer, high prevalence of complex inflammatory skin diseases, and increasing desire for cometic procedures have also contributed to an increased demand for dermatologists that is expected to continue to increase for the foreseeable future [9,14,15,16,17,18,19].

Additionally, the need for virtual care became especially evident with the COVID-19 pandemic. The large scale of the pandemic has caused healthcare systems to shift how they deliver care quickly and substantially [20]. The number of in-person visits to ambulatory practices in the US declined nearly 60% in early April 2020, and although that number is rising, it is still lower than pre-pandemic levels [21]. Dermatology had one of the highest cumulative declines in visits, including both in-person and virtual, at a 22% decrease from baseline [21]. With the current threat of the delta and omicron variants and the realized potential of future pandemics, the ability to provide high-quality care virtually will likely remain an important part of medicine.

Telehealth platforms available through smartphone apps are especially important for increasing access to care for patients, and many patients are open to the use of teledermatology services [22,23,24]. Teledermatology has proven to be comparable to conventional clinic-based care concerning clinical outcomes and patient satisfaction [25]. By reducing the number of in-person visits, teledermatology saves on costs associated with travel and workplace absenteeism [25,26,27]. Additionally, teledermatology can be a faster method to deliver care, as it has been shown to decreases appointment waiting times and total consultation times [27,28]. While mobile teledermatology has been proven to be a reliable way to diagnose skin conditions, a systematic review by Clark et al. reported that diagnostic accuracy of traditional in-person dermatology was superior to mobile teledermatology, with a weighted mean absolute difference of 7.2% [29]. Of note, the two studies included in this review that reported on diagnostic accuracy were from 2011 and 2013 and reported on different primary measures [30,31]. In a more recent prospective diagnostic study, mobile teledermatology successfully detected all cases of skin cancer in a low prevalence population [32]. Furthermore, the addition of dermoscopic images to conventional images resulted in even higher specificity (85% vs. 77%), preventing unnecessary further testing of benign lesions. Multiple studies have also reported on the benefit of mobile teledermatology as a triage system where patients with skin lesions suspicious for cancer can be prioritized and referred to an in-person dermatologist for further evaluation [33,34].

For users without easy access to dermatologists, the ability to self-monitor is an especially important function provided by some apps. Utilizing apps with AI to analyze suspicious lesions may prompt them to make a telehealth appointment or seek out an in-person dermatologist. Many of these apps offer the option to speak with a dermatologist through the app. Apps that allow users to photograph and monitor skin lesions over time provide an organized way for users to look for development of concerning features and provide pictures for dermatologists to review during appointments. There is an added advantage when patients use dermatoscope attachments for their smartphones, as dermoscopy has been shown to improve diagnostic accuracy [35,36]. Studies have also shown that users find mobile teledermoscopy to be easy to conduct and reinforces the importance of conducting self-skin examinations [35,37]. Apps that focus on disease monitoring may help users more easily identify triggers causing flare ups, especially apps that factor in diet, stress level, weather, and allergens such as pollen and mold. These apps may help users feel more confident self-monitoring their skin lesions between dermatologist visits.

Smartphones and medical apps are becoming increasingly more relied on by physicians, residents, and health educators in medical practice [38]. A survey of dermatologists in India reported that most respondents preferred using smartphones for clinical photography over conventional digital cameras [39]. While most clinical photos taken by dermatologists are for disease monitoring, dermatologists also take photos for additional reasons including teaching, research, and consulting with other physicians [39,40]. A recent survey of dermatologists and trainees in Australia reported that the majority, especially junior practitioners, sent and received photographs from their smartphone on a regular basis, with 82% of them rating this ability as very important [41]. Smartphones are typically smaller and more portable than conventional digital cameras. Recent advances in smartphone cameras have allowed for photography in a variety of lighting and with lossless optical zooming. One of the main advantages of using smartphones for photos is the ease in capturing and transferring images [42]. Additionally, smartphones also have the ability to easily record and transfer videos, which may be especially useful for recording dermatologic procedures for teaching purposes. Importantly, studies have shown that patients are generally accepting of medical photography, including having images recorded on a physician’s personal smartphone, as long as they are aware of the security measures taken to ensure privacy [43,44].

General dermatology reference and educational apps give physicians easy access to comprehensive information related to dermatologic conditions and can help them stay up to date with evidence-based practices. A recent survey of 210 dermatologists in Kuwait reported that 94.1% used smartphones to access medical information, with 68.3% using smartphones for this purpose on a daily basis [45]. Many physicians in dermatology residency programs have expressed interest in having digital dermatology tools incorporated more into resident training. Smartphones can also be used to evaluate the efficacy of residency training programs [46]. For example, a dermoscopy curriculum referred to as Dermatology Early Melanoma Detection (DERM:EMD), was recently developed by 3 dermatologists in the US and incorporated into 8 dermatology residency programs. Using smartphone-enabled survey tools such as Kahoot! And Qualtrics, they were able to gather metrics on individual participant, as well as institutional and program, performance [47]. Educational apps that incorporate clinical, dermoscopy, and pathology images are especially useful in the field of dermatology, a largely visual-based specialty, and can be very useful portable study tools for resident physicians preparing for their board examinations [48]. Additionally, apps that provide quizzes and challenges in the form of games provide engaging ways for physicians to study material. These types of educational games have been termed “serious games”, as they are games that do not have entertainment as the primary purpose but are designed to engage players for the main purpose to educate or train [49,50]. While this is still an emerging field, studies have shown that the use of serious games for health professions education is efficacious for short-term learning and may even be more efficacious than conventional methods [51,52].

### 4.2. Challenges and Limitations

Despite the plethora of health-related apps available to the public, there is a significant lack of high-quality studies on the quality, accuracy, and safety of these apps [53]. The peer-reviewed studies that have been done have shown variable quality and accuracy of available apps [4,54,55,56]. For example, a recent systematic review of apps available for eczema found that approximately one third of apps provided misleading information regarding aspects such as treatment and disease progression and less than one sixth provided international guideline-supported information on therapies [56]. Another review of apps available for acne found that the majority of the included apps had Masud scores less than 15, with scores from 5–10 indicating apps not likely to be beneficial and may even be harmful and scores from 11–15 indicating apps that may be beneficial but likely have shortcomings [55]. Lastly, a review of skin cancer apps found that less than half of the apps self-reported input from clinician or professional bodies and less than one tenth reported peer-reviewed evidence of their efficacy [4]. This study also reported that many educational skin cancer apps do not appear to be regularly updated and may contain outdated information regarding guidelines for the prevention, diagnosis, and management of melanoma and other skin cancers [4].

With the incorporation of AI technology into apps available to the public, there are concerns about accuracy and reliability, especially when analyzing suspicious skin lesions for features of skin cancer. A systematic review in 2020 analyzing the accuracy of six algorithm-based apps for skin cancer detection determined that the available apps could not be relied on to detect all cases of melanoma or other skin cancers [54]. The app with the highest reported accuracy for detecting features concerning melanoma had a sensitivity of 88% and a specificity of 79% [57]. Some users have also noted inconsistent results when re-imaging the same lesion in reviews. The quality of the images used for analysis depends on factors such as zoom, lighting, and angle, and variations in these may affect results. Inaccuracies may lead to false reassurance for concerning lesions or unnecessary worry for benign lesions, despite disclaimers on the apps that the results should only be used as a guide and cannot replace healthcare advice [54]. However, these algorithms may one day be improved to the point of becoming reliable screening tools [54]. Studies using AI algorithms to classify clinical and dermoscopic images of skin lesions as benign or malignant have shown diagnostic accuracy comparable to those of dermatologists, highlighting the potential of this technology [58,59]. For AI to become a useful tool in this context, inaccuracies will need to be addressed and providers will need to educate patients on the limitations of AI technology. Additionally, AI is commonly used in dermatology-related apps for analysis of skin features to provide product recommendations. Some these apps, such as MDacne and Acne Intelligence, create personalized treatment regimens using their own products. Users should be aware of the possible influence of pharmaceutical companies and skin care brands, especially when products are being recommended.

Lack of regulation is another significant concern related to health-related apps [60]. In 2013, the FDA first issued the Policy for Device Software Functions and Mobile Medical Applications Guidance, explaining the administration’s oversight of medical mobile apps [61]. In 2019, this policy was updated to clarify that their focus is on function, regardless of the platform, and changed “mobile application” to “software function” in the policy. The policy states that the FDA applies the same risk-based approach to software functions as they do to other medical devices, and any software that poses a great risk to patients will require FDA review. Software functions that require review are those that “may be intended for use in the diagnosis of disease or other conditions, or in the cure, mitigation, treatment, or prevention of disease” [61]. Examples of app functions that do not require review include educational apps designed for healthcare providers and apps for general patient education with the goal to increase patient education and empowerment but not replace the direction of a health professional or perform clinical assessment. However, no skin cancer detection apps have received FDA approval to date, yet many remain available to download, raising the question of the efficacy of this policy in regulating medical mobile apps.

Patient safety and security also needs to be taken into consideration when using smartphones. Providers can be at risk of violating the Health Insurance Portability and Accountability Act (HIPAA), especially when handling clinical photos. In addition to the possibility of losing a device or having it stolen, issues such as non-encrypted communications and phone hacks are also a concern [62]. A survey of dermatologists in the United States reported that nearly half of providers who stored patient photos on their personal smartphones did not have them secured or encrypted [63]. Additionally, many smartphones have the option to automatically upload photos to a cloud, which also can be subject to security breaches [62]. One way to mitigate the risk of HIPAA violation is to transfer photos through HIPAA-compliant messaging services, such as those available through (Electronic Health Records) EHR systems or email platforms [62]. Additionally, patient photos should also be stored in a secure, HIPAA-compliant location, such as on the patient’s EHR or HIPPA-compliant clouds [62].

Another drawback of smartphones for dermatology is cost. Aside from the costs associated with purchasing a smartphone and paying for service, many apps available also have some cost associated, even if they are free to download. In this study, only costs to download were assessed and most apps were free to download. Download fees for the remaining apps ranged from USD 0.99 to USD 349.99. Apps may have a one-time purchase fee, require institutional access or a subscription, or be pay per service [4]. A review of apps for skin cancer detection in 2019 reported that one-time purchase fees ranged from USD 0.99 to USD 15.99, 12-month subscriptions ranged from USD 3.82 to USD 34.99, and the most expensive price for a dermatologic consult was USD 399.96 [4]. Sometimes apps are only free to a certain capacity; for example, some of the mole-tracking apps only allow users to add a few images for free before requiring them to pay. Other apps may require users to pay to hide advertisements [64]. Additionally, if users are recommended to consult with an affiliated dermatologist through the app, they may end up paying more money, especially if the consulting dermatologist recommends following up with an in-person dermatologist.

## 5. Limitations

The present study only assesses apps available in the Apple App Store and Google Play Store from the United States. Additionally, only apps with an English option available were included. Therefore, our study likely did not capture all dermatology-related apps available worldwide, and some of the apps discussed may not be available outside of the United States. This may affect the generalizability of the findings reported here.

## 6. Conclusions

Given the increasingly widespread use of smartphones, they have the potential to become an important tool in dermatology, especially to reach underserved populations and limit contact during the COVID-19 pandemic. Many of the diverse functions that can be carried out from a smartphone are in the form of apps designed for both patients and providers. However, there is a need for peer-reviewed studies and stricter regulation to ensure that available apps are accurate, reliable, and up to date. Notably, there was a dearth of studies evaluating the use of apps for UV protection. Given that there are over 60 apps available in this category, it is important for future studies to begin exploring the use of these apps. Health-related apps are likely to continue to increase in number and experience high turnover, and it is important for healthcare providers to keep up with health-related functions available on smartphones and inform patients about the benefits and limitations of available apps.

## Figures and Tables

**Table 1:** Characteristics of the top apps per category based on number of reviews for categories with 50 or more apps.

Category	Name	No. of Reviews	Platform	Download Cost (USD)	Target Audience	AI?
Disease guide	MDacne—Custom Acne Treatment	16,971	Apple, Android	0	Patients	Yes
	Kopa by Happify Health	432	Apple, Android	0	Patients	No
	Acne Intelligence	270	Apple, Android	0	Patients	Yes
	Imagine Skin Condition Tracker	248	Apple, Android	0	Patients	No
	myForte	135	Apple, Android	0	Patients	No
Self-surveillance/ diagnosis	TroveSkin—Get Clearer Skin	84,329	Apple, Android	0	Patients	No
	Medgic—Scan, Analyze and Detect Skin Problems	4895	Android	0	Patients	Yes
	Skin Bliss: Cosmetics & Beauty	2171	Apple, Android	0	Patients	Yes
	Miiskin—Skin Cancer eHealth	2456	Apple, Android	0	Patients	Yes
	Skincare routine	1132	Apple, Android	0	Patients	No
Teledermatology	Teladoc|Telehealth & Therapy	423,114	Android	0	Patients	No
	Practo: Online Doctor Consultations & Appointments	216,103	Apple, Android	0	Patients	No
	MDLIVE: Talk to a Doctor 24/7	66,352	Apple, Android	0	Patients	No
	Lybrate: Consult Doctor Online	66,165	Apple, Android	0	Patients	No
	Zocdoc	18,970	Apple, Android	0	Patients	No
General dermatology reference	VisualDx	564	Apple, Android	0	Providers	Yes
	Dermatology Atlas & Skin Infections	399	Android	0	Providers	No
	Skin Disease and Treatment (a)	232	Android	0	Patients, Providers	No
	Skin Diseases and Treatment (b)	195	Android	0	Patients, Providers	No
	Skin Disease Treatment Symptoms and Diagnosis 2019	123	Apple, Android	0	Patients, Providers	No
Ultraviolet (UV) protection	Weather data & microclimate: Weather Underground	477,067	Android	0	Patients	No
	Weather Home—Live Radar Alerts & Widget	118,896	Android	0	Patients	No
	UV index widget—worldwide	5180	Apple	0	Patients	No
	UV Index Now Forecast & Sun Tracker—UVI Mate	2020	Apple, Android	0	Patients	No
	UVLens—UV Index	1456	Apple, Android	0	Patients	No
Educational aid	Dermatology by Dr. Manish Soni	161	Apple, Android	0	Providers	No
	Dermoscopy Two Step Algorithm	128	Apple, Android	0	Providers	No
	iDoc Academy	113	Apple, Android	0	Providers	No
	Skin Anatomy	79	Android	0	Providers	No
	Top Derm: A game for dermatologists	73	Apple, Android	0	Providers	No

(a) Developed by Jankari; (b) Developed by Adria Devs.

**Table 2:** Characteristics of the top apps per category based on number of reviews for categories with less than 50 apps.

Category	Name	No. of Reviews	Platform	Download Cost (USD)	Target Audience	AI?
Conference	AAD Meetings	22	Apple, Android	0	Providers	No
	Dermacon 2020	12	Apple, Android	0	Providers	No
	Fall Clinical	3	Apple, Android	0	Providers	No
Calculator	Melanoma Calculator	13	Apple, Android	0	Providers	No
	Skin Lymphoma	8	Apple	0	Providers	No
Photography	Sklip	12	Android	0	Patients	Yes
	HumazeMD	8	Apple, Android	0	Providers	No
Research	PeDRA Research	24	Apple, Android	0	Providers	No
Other	Think Dirty	46,150	Apple, Android	0	Patients	No
	SkinSafe	178	Apple, Android	0	Patients	No
	EZDERM	38	Apple, Android	0	Patients	No

## Data Availability

Data sharing not applicable.

## Appendix A

**Table A1:** Developers of the apps mentioned in the review.

Name	Developer	Address
MDacne—Custom Acne Treatment	MDalgorithms Inc.	548 Market St. #86774 San Francisco, CA 94104, USA
Kopa by Happify Health	Happify, Inc.	51 E 12th St. 5th Fl, New York, NY 10003, USA
Acne Intelligence	SkinClinical AI, LLC	2000 N Racine Ave Ste 3100 Chicago, IL 60614, USA
Imagine Skin Condition Tracker	LEO Pharma A/S	Industriparken 55 Ballerup, 2750 Denmark
myForte	Galderma Laboratories, L.P.	14501 North Fwy Fort Worth, TX 76177, USA
TroveSkin—Get Clearer Skin	TROVE TECHNOLOGIES PTE. Ltd.	1015 Geylang E Ave 3 #01-131 Geylang East Industrial Estate Singapore (389730)
Medgic—Scan, Analyze and Detect Skin Problems	MEDNET PTE Ltd.	710 Bedok Reservoir Rd #04-3120 Bedok Reservoir Garden Singapore (470710)
Skin Bliss: Cosmetics & Beauty	Skin Bliss SIA	Rīga, Matīsa iela 61A—19, LV-1009 Latvia
Miiskin—Skin Cancer eHealth	Miiskin ApS	Fruebjergvej 3 2100, København Ø, Hovedstaden Denmark
Skincare routine	Mento Apps Ltd.	23 Stoneleigh Avenue, Brighton BN1 8NP, UK
Teladoc|Telehealth & Therapy	Teladoc, Inc.	2 Manhattanville Road Suite 203 Purchase, New York, NY 10577, USA
Practo: Online Doctor Consultations & Appointments	PRACTO TECHNOLOGIES PRIVATE Ltd.	Vedanta Cts, 401, No 779, Mumbai City Makhwana Road Marol Andher Mumbai, Maharashtra, 400059 India
MDLIVE: Talk to a Doctor 24/7	MDLIVE, Inc.	13630 NW 8th Street Suite 205 Sunrise, Miramar, FL 33325, USA
Lybrate: Consult Doctor Online	Lybrate, Inc.	340 South Lemon Avenue Suite 7794 Walnut, CA 91789, USA
Zocdoc	Zocdoc, Inc.	568 Broadway Floor 2 New York, NY 10012, USA
VisualDx	Logical Images, Inc.	3445 Winton Place Suite 240 Rochester, NY 14623, USA
Dermatology Atlas & Skin Infections	Medico_guide	Plot 542 Mahnti House, Surya Nagar, Bhubaneshwar 751003 India
Skin Disease and Treatment	Jankari	Not available
Skin Diseases and Treatment	Adria Devs	India (Full address not available)
Skin Disease Treatment Symptoms and Diagnosis 2019	Nuril Development Team	Not available
Weather data & microclimate: Weather Underground	Weather Underground, LLC	300 Interstate North Pkwy SE Atlanta, GA 30339-2403, USA
Weather Home—Live Radar Alerts & Widget	Position Mobile Ltd. SEZC	90 North Church St. Strathvale House, George Town, Cayman Islands, KY1-9012
UV index widget – worldwide	Bjorn Jenssen	Not available
UV Index Now Forecast & Sun Tracker—UVI Mate	Full Stack Cafe Pty Ltd.	Not available
UVLens—UV Index	Spark 64 Limited	80 Queen Street, Auckland Central, Auckland, 1010, New Zealand
Dermatology by Dr. Manish Soni	Prepladder Pvt Ltd.	Plot A 12, IT Park Chandigarh, Punjab, 160101 India
Dermoscopy Two Step Algorithm	Usatine & Erickson Media LLC	8529 Raintree Woods Dr, Fair Oaks Ranch, TX 78015, USA
iDoc Academy	iDoc Academy	4113 Saint Charles Rd Bellwood, IL 60104, USA
Skin Anatomy	Visual 3D Science	8821 Midway West Rd Raleigh, NC 27617, USA
Top Derm: A game for dermatologists	Level Ex, Inc.	180 N La Salle St. Ste 500 Chicago, IL 60601, USA
Epic Haikuu	Epic Systems Corporation	1979 Milky Way Verona, WI 53593, USA
Kahoot!	Kahoot! AS	Fridtjof Nansens Plass 7 Oslo, 0160 Norway
Qualtrics	Qualtrics Labs, Inc.	333 River Park Dr Provo, UT 84604, USA
AAD Meetings	American Academy of Dermatology	9500 W Bryn Mawr Ave, Suite 500 Rosemont, IL 60018, USA
Dernacon 2020	Dermacon2020	B-305, Sarita, Prabhat Industrial Estate, Dahisar East, Mumbai 400068 India
Fall Clinical	Foundation for Research and Education in Dermatology, LLC	2121 N Front Age Rd W #253 Vail, CO 81657, USA
Melanoma Calculator	Doorn Corporation	2902 Meadow Farms Pl Louisville, KY 40245, USA
Skin Lymphoma	Integrated Cancer Research Limited	206 Upper Richmond Road West, East Sheen, London SW14 8AH, UK
Sklip	Sklip Inc.	Not Available
HumazeMD	HumazeMD, LLC	3054 Woodwalk Dr SE Atlanta, GA 30339, USA
PeDRA Research	Pediatric Dermatology Research Alliance, Inc.	205 SE Spokane St, Ste 300, Portland, OR 97202, USA
Think Dirty	Think Dirty Inc.	341 King St. E Suite 617 Toronto, ON M5A 1L1, Canada
SkinSafe	HER Inc.	3104 E Camelback Road #726 Phoenix, AZ 85016, USA
EZDERM	EZDERM, LLC	2640 Golden Gate Pkwy Ste 201 Naples, FL 34105, USA
